# Supplementary material for: Thermal assisted self-organization of calcium carbonate
Source: Nat Commun. 2018 Dec 6;9:5221. doi: 10.1038/s41467-018-07658-0 (PMC6283884; doi:10.1038/s41467-018-07658-0)
Supplement: Supplementary file 1 — Supplementary Information [file 41467_2018_7658_MOESM1_ESM.pdf]

Supplementary Information for

Thermal assisted self-organization of calcium carbonate

Zhang et al.

This file includes:

Supplementary Figures 1 to Figure 10, and the references to the Supplementary Information.

1. Solubility of calcium carbonate/MHC and silica versus temperature

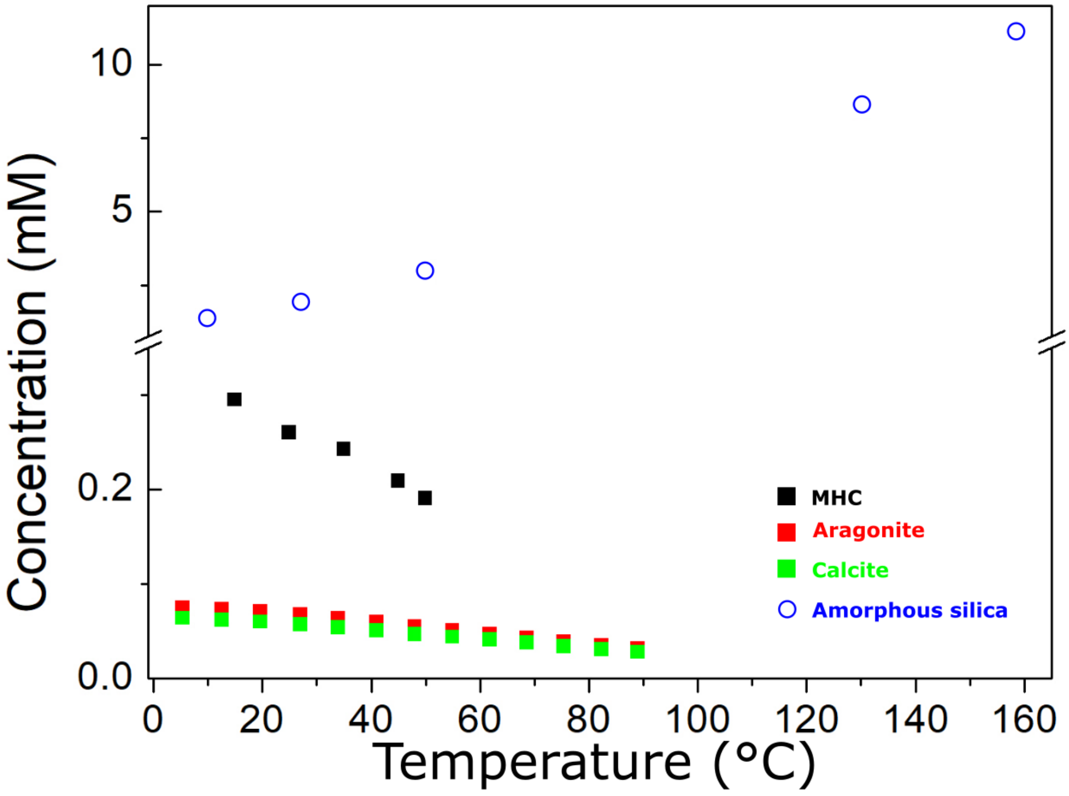

Supplementary Figure 1. Solubility of silica and calcium carbonate versus temperature from 0 °C to 160 °C. The solubility data of amorphous silica are from the works of Hitchen and Kitahara at alkaline conditions [1,2]; the solubility data of MHC are from the work of Kralj et al. at the pH range from 9.7 to 10.5 [3]; and the solubility data of aragonite and calcite are from the work of Plummer et al.[4]

2. Crystallization of monohydrocalcite in alkaline silica gel by counterdiffusion method

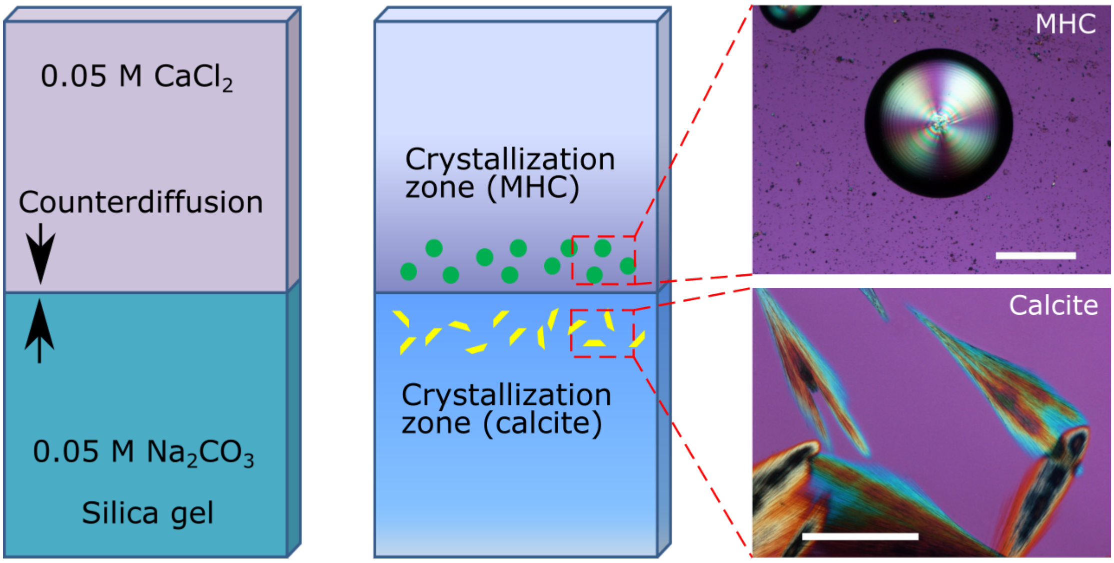

Supplementary Figure 2. Scheme of the crystallization of monohydrocalcite by counterdiffusion method. The crystallization is performed in alkaline silica-rich solution in a glass cassette by the counterdiffusion method and the optical micrographs depicting different precipitates formed at room temperature (hemispherical monohydrocalcite in liquid and the elongated sheaf-of-wheat shaped calcite in gel). Scale bar: 200 µm.

3. Characterization of monohydrocalcite at different temperatures

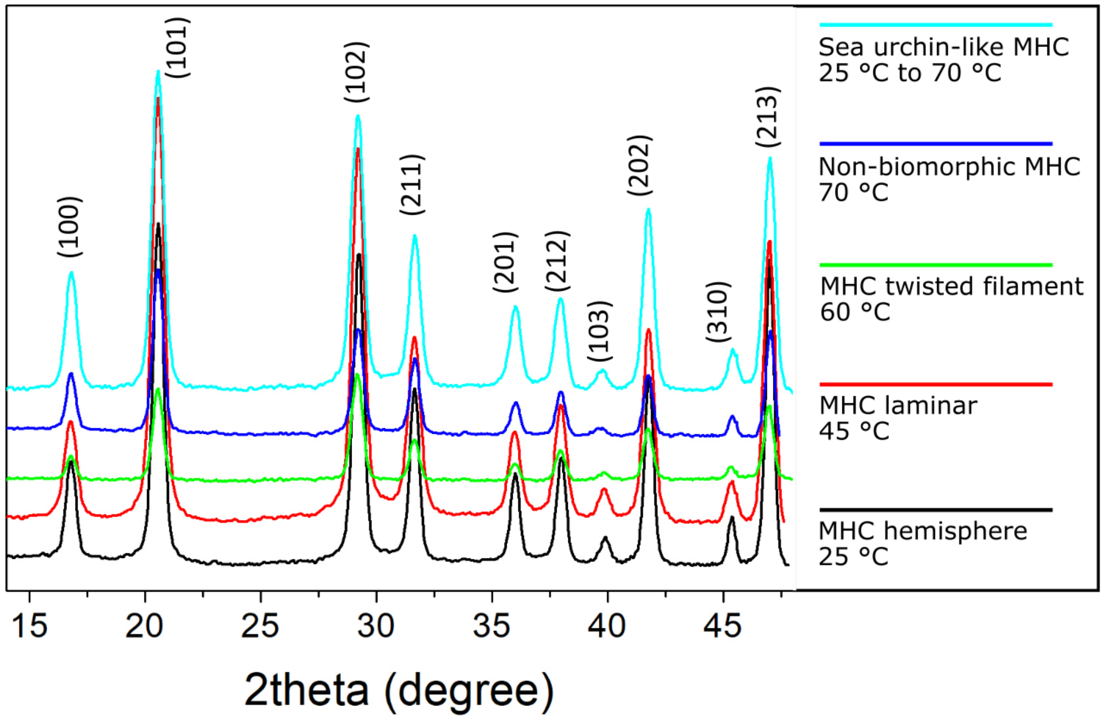

Supplementary Figure 3. X-ray diffraction patterns of the monohydrocalcite form at different temperatures (25, 45, 60 and 70 °C), and the sea urchin-like monohydrocalcite form by the manner of stepwise adjustment of temperature on growth (25 °C to 70 °C). All the peaks correspond to the characteristic family planes of the monohydrocalcite structure.[5-7]

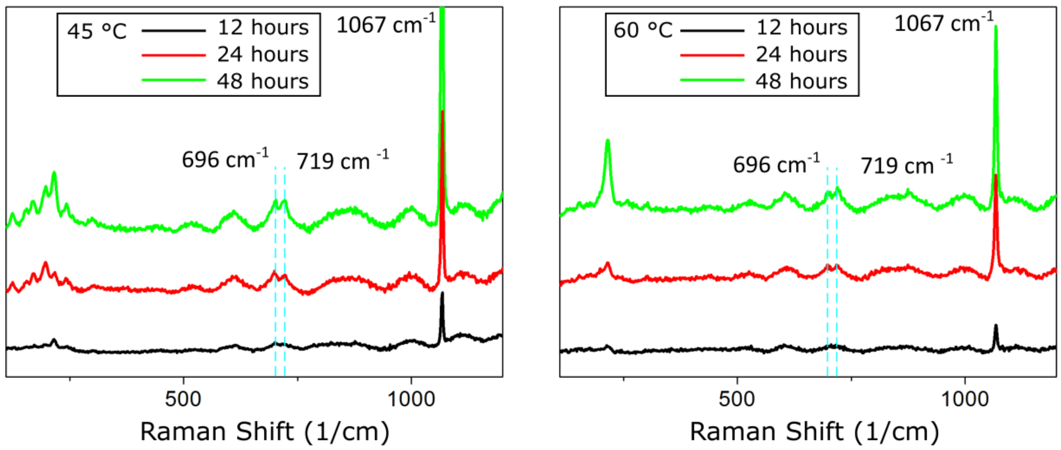

Supplementary Figure 4. Raman spectra from the time-lapse in situ measurements on the monohydrocalcite with different morphologies: flower-like particles form at 45 °C and curvilinear sheet with twisted filament form at 60 °C. The corresponding peaks of MHC (696 cm^{-1}, 719 cm^{-1} and 1067 cm^{-1})[8] are labeled.

4. Supplementary discussion 1: Morphogenesis of biomorphic monohydrocalcite

The laminar sheets are commonly observed at the early stages of the growth of biomorphic monohydrocalcite at 45 and 60 °C. In some special cases, the laminar sheets evolve into branches along certain directions from the rim of the previous laminar sheets (Supplementary Fig. 5 a). The shape of each branch is actually a sheet rolled on itself, controlled by three main growing vectors (marked by arrows with different colors in Supplementary Fig. 5 b, c). The elongation and width of the branch is marked by growth along V_1 (red arrow in Supplementary Fig. 5 b) and V_2 (green arrow in Supplementary Fig. 5 b). The growth of V_3 (blue arrow in Supplementary Fig. 5 b) occurs perpendicular to V_1 and varying angle with respect to V_2. As shown in Supplementary Fig. 5, this angle leads to curling along the rim of the branch (colored by yellow in Supplementary Fig. 5 c), resulting an irregularly curvilinear laminar shape. The level of curling is controlled by the V_2-V_3 angle and the velocity of V_2 and V_3, and the elongation of the laminar sheet is controlled by V_1 (Supplementary Fig. 5 c).

At higher temperature (60 °C), growth along V_2 is decelerated, causing branches to develop into filaments along V_1 directions in case growth along V_1 becomes

significantly faster than along V_2 (Panel B in Supplementary Fig. 5). However, if growth along V_3 is faster than along V_1, the filament grows twisted and leads to the continuous curling, which further produces a bamboo-like joint in the twisted position (Supplementary Fig. 5 e, f). The growth of these twisted filaments seems to provoke similar morphogenesis to the worm-like braids of barium carbonate biomorphs. Since only a few cases show $V_3 > V_1$, a sparse amount of bamboo-like joints appear in the filaments. These joints appear in the FESEM analysis indicating that they are constructed by plenty of co-oriented rod-like subunits (Supplementary Fig. 5 e). In the case when $V_2 > V_3$, the level of curling decreases and a new laminar sheet forms by the growth along V_2 (Supplementary Fig. 5 h). In addition, there is also the case at 45 °C when $V_2 \approx V_3 > V_1$. In this case, the continuous curling induces rhythmic twists along the growth of V_1, which produces a twisted ribbon like laminar sheet (Supplementary Fig. 5 i). This behavior is also similar to the morphogenesis of barium carbonate biomorphs.

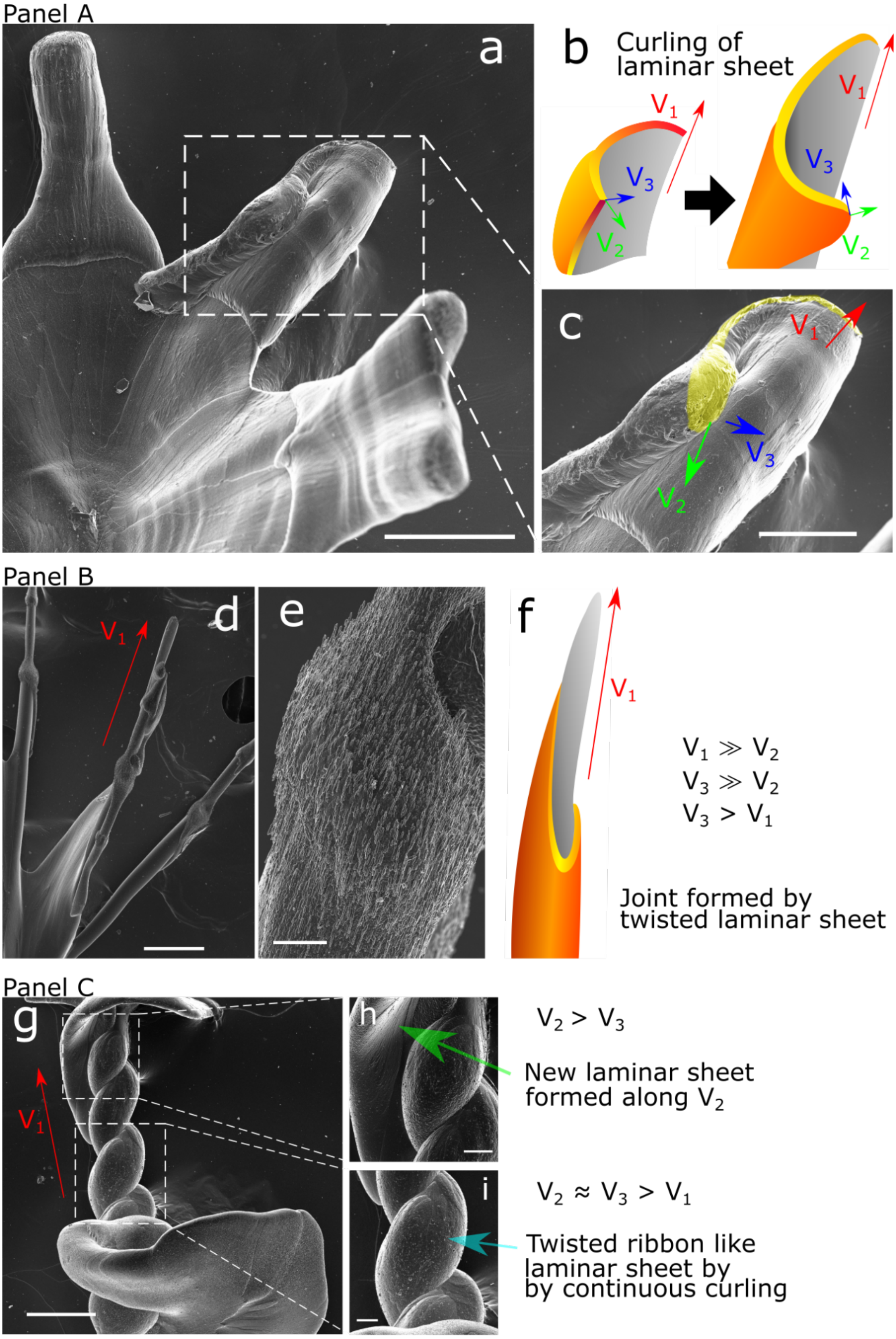

Supplementary Figure 5. FESEM images and scheme of the morphogenesis of the biomorphic monohydrocalcite form at 45 and 60 °C. FESEM image of the developed of

braches from laminar sheet (a) and the close-up view of the curling of the branch (b); scheme of the growth model of the curling of a laminar sheet controlled by different growth vectors (c); FESEM images of the filaments develop from laminar sheets formed at 60 °C (d), and the closed-up view of the bamboo-like joint (e) in the twisted position of filament as well as the scheme of growth (f); FESEM image of the twisted ribbon like laminar sheet form at 45 °C (g) and the closed-up views of the new developed laminar sheet (h) and the position of twist (i). Scale bars: 100 μm (a, g), 50 μm (b), 200 μm (d), 10 μm (e), 20 μm (h, i).

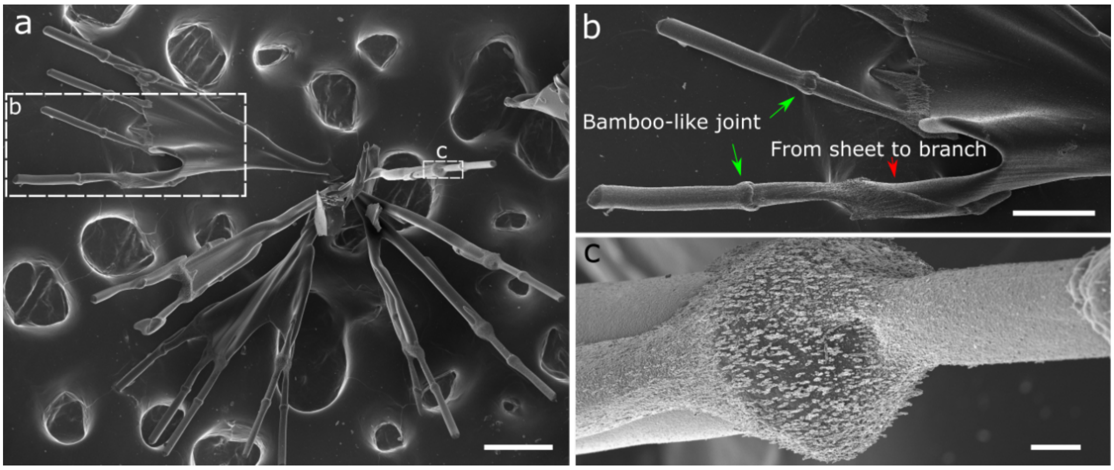

Supplementary Figure 6. FESEM images of curving laminar sheets with bamboo-like joints formed at 60 °C. The general view of the MHC structure is shown in (a); the transition from laminar sheet to branch is shown in (b), the location where the curing occurred is marked by red arrow, and the bamboo-like joints are marked by green arrows; the close-up view of the bamboo-like joint formed along the branch is shown in (c). The morphogenesis of the branches and bamboo-like joints are shown in Supplementary Fig. 5 above. Scale bars: 200 μm (a), 100 μm (b), 10 μm (c).

5. Supplementary discussion 2: Fabrication of heterotextured complexity by temperature control

By the counterdiffusion method described above, hemispherical monohydrocalcite particles were firstly produced in the glass cassette at 25 °C, and then the cassette was transported to the oven at 70 °C for about 12 hours growth. As a result, the MHC particles experienced the different growth stages at 25 °C, the temperature transition from 25 °C to 70 °C, and then 70 °C. The change of the temperatures led to different textures as well as the variation of Ca/Si ratio at the corresponding positions (Supplementary Fig. 7, particle was collected after 30 min after it was transported to the oven at 70 °C). These textures include the spherulite formed at 25 °C, the thin layer formed during the transition of temperature and the polycrystalline clusters formed at 70 °C. The EDX spectra show the decreasing tendency of Ca/Si ratio by increasing temperature.

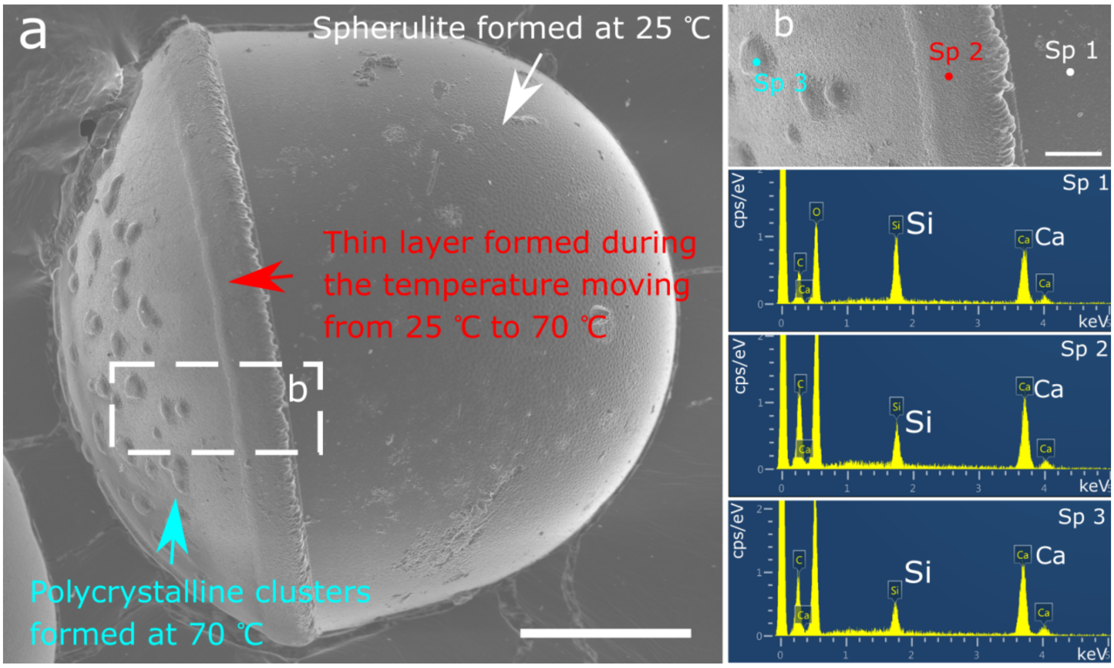

Supplementary Figure 7. The growth process of MHC from 25 °C to 70 °C and the EDX spectra at different textures from the corresponding growth stages. The general view of the growing heterotextured MHC is shown in (a), the corresponding textures are labeled by white (25 °C), red (transition from 25 °C to 70 °C), and cyan (70 °C) in turn. The close-up view (b) shows the area including these three different textures and the sites for the EDX analysis are also colored by white (Sp1, 25 °C), red (Sp2, transition from 25 °C to 70 °C) and cyan (70 °C). Scale bars: 50 μm(a), 10 μm (b).

The thin layers covered the spherulite by increasing the temperature (from 25 °C to 70 °C), and the polycrystalline cluster grew at 70 °C and further developed into faceted sticks on the surface of the thin layers (Supplementary Fig. 8 Panel A), which made the entire heterotextured MHC exhibit sea urchin-like shape (Supplementary Fig. 8 d and Fig. 9). The close-up views indicate that both, the thin layer and the polycrystalline clusters are composed of nanodrops (Supplementary Fig. 8 Panel B), but when the clusters developed into the faceted sticks, the crystallographic symmetry are preserved (Supplementary Fig. 9 b).

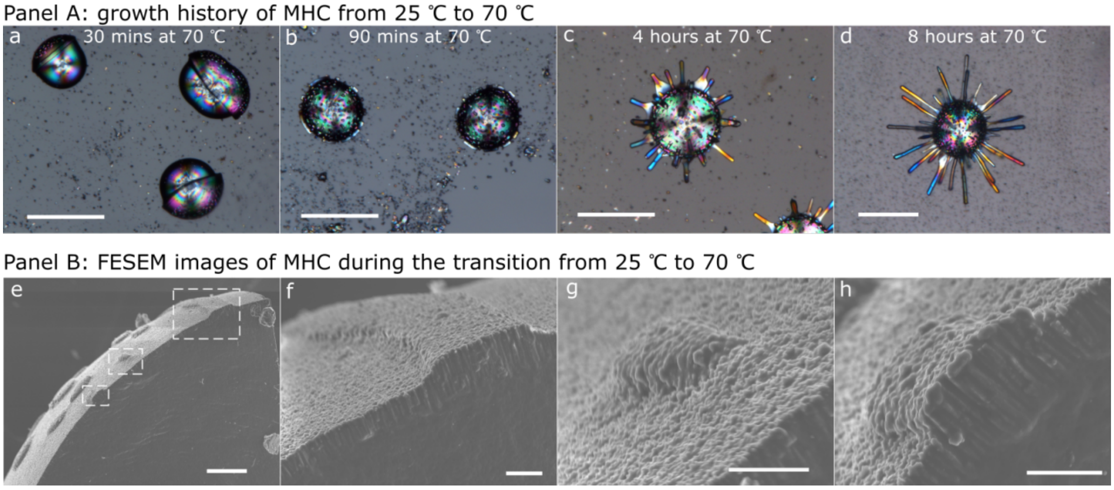

Supplementary Figure 8. Panel A: Optical micrographs showing the growth history upon heating from 25 °C to 70 °C (a) and then at constant 70 °C temperature (b, c, d); Panel B: FESEM images of MHC during the transition from 25 °C to 70 °C, the textures of the thin layer and the polycrystalline cluster are shown in close-up views (f, g, h). Scale bars: 100 μm (a, b, c, d), 10 μm (e), 2 μm (f, g, h).

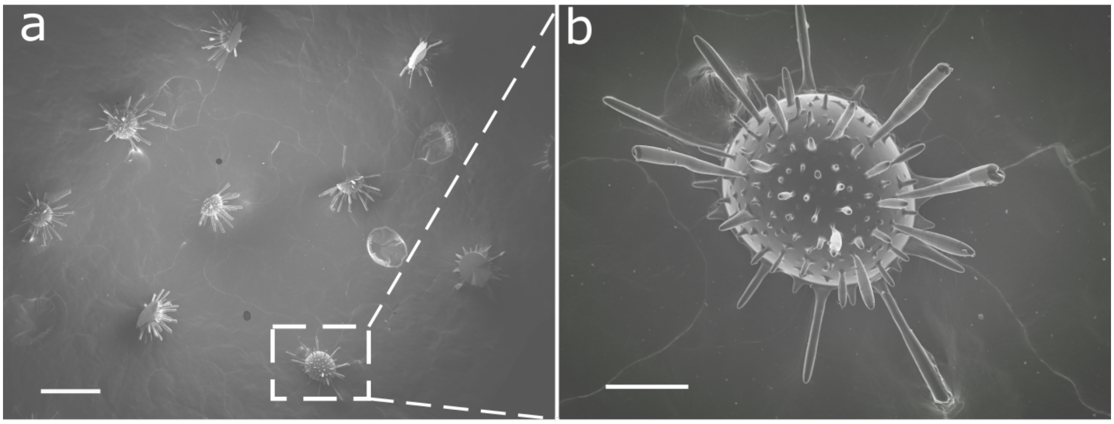

Supplementary Figure 9. The overview FESEM image of the sea urchin-like heterotextured monohydrocalcite formed by the stepwise adjustment of temperature from 25 °C to 70 °C (a), and the close-up view of a selected heterotextued monohydrocalcite exhibiting facets on the top of the sticks (b). Scale bar: 500 μm (a), 100 μm (b).

Heterotextured complex morphologies of monohydrocalcite were fabricated by the more complicated modulations of temperature (Supplementary Fig. 10). The formed complex motphologies experienced different stepwise adjustments of temperature in

turn: 48 hours at 25 °C, 4 hours at 70 °C, 20 hours at 45 °C, 24 hours at 60 °C and 24 hours at 70 °C again.

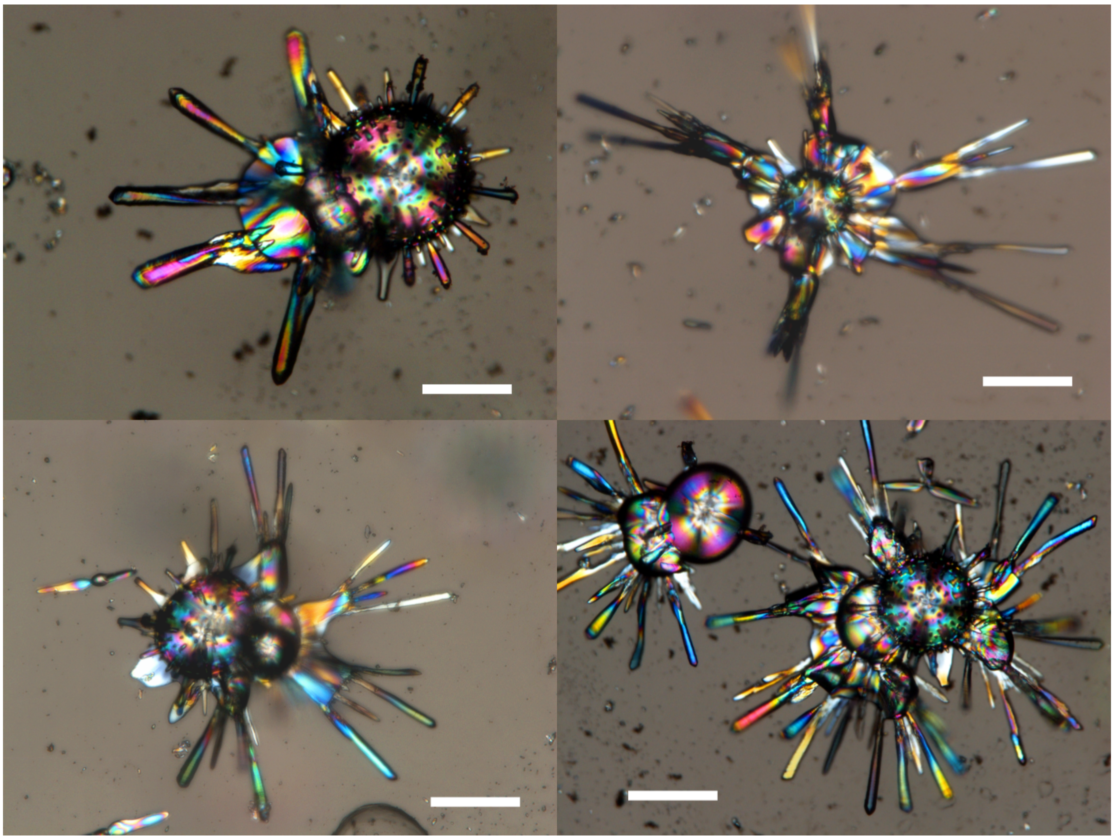

Supplementary Figure 10. Optical micrographs (by polarized light) of the heterotextured complex morphologies of monohydrocalcite formed at sequential stepwise adjustments of temperature. Scale bar: 200 μm

References

1 Hitchen, C. S. Method for the experimental investigation of hydrothermal solutions, with notes on its application to the solubility of silica. *Bull. Inst. Min., Met.; ()*, Medium: X; Size: Pages: 1-34, 29-32 (1935).

2 Kitahara, S. The polymerization on silicic acid obtained by the hydrothermal treatment of quartz and the solubility of amorphous silica. *Review of physical chemistry of Japan* **30**, 131-137 (1960).

3 Kralj, D. & Brečević, L. Dissolution kinetics and solubility of calcium carbonate monohydrate. *Colloids and Surfaces A: Physicochemical and Engineering Aspects* **96**, 287-293, (1995).

4 Plummer, L. N. & Busenberg, E. The solubilities of calcite, aragonite and vaterite in CO_2-H_2O solutions between 0 and 90°C, and an evaluation of the aqueous model for the system $CaCO_3$-CO_2-H_2O. *Geochimica et Cosmochimica Acta* **46**, 1011-1040, (1982).

5 Lueger-Ging, K. & Effenberger, H. in *European Crystallographic Meeting.* 107.

6 Effenberger, H. Kristallstruktur und Infrarot-Absorptionsspektrum von synthetischem Monohydrocalcit, $CaCO_3 \cdot H_2O$. *Monatshefte für Chemie* **112**, 899-909, (1981).

7 Zhang, G., Delgado-Lopez, J. M., Choquesillo-Lazarte, D. & Garcia-Ruiz, J. M. Crystallization of monohydrocalcite in a silica-rich alkaline solution. *CrystEngComm* **15**, 6526-6532, (2013).

8 Tlili, M. M. *et al.* Characterization of $CaCO_3$ hydrates by micro-Raman spectroscopy. *Journal of Raman Spectroscopy* **33**, 10-16, (2002).
